# Lung ultrasound findings in patients with COVID-19 pneumonia

**DOI:** 10.1186/s13054-020-02876-9

**Published:** 2020-04-28

**Authors:** Changyang Xing, Qiaoying Li, Hong Du, Wenzhen Kang, Jianqi Lian, Lijun Yuan

**Affiliations:** 1 Department of Ultrasound Diagnostics, Tangdu Hospital, Fourth Military Medical University, Xi’an, 710038 China; 2 Department of Infectious Diseases, Tangdu Hospital, Fourth Military Medical University, Xi’an, 710038 Shaanxi China

**Keywords:** COVID-19, Lung ultrasound, SARS-CoV-2, Deep vein thrombosis

Since December 2019, the outbreak of pneumonia caused by a new coronavirus [1], which was later identified as coronavirus disease 2019 (COVID­19), has infected more than 410,000 patients globally according to the situation report of World Health Organization. Lung ultrasound is an important tool for the diagnosis and follow-up of pneumonia in neonates, children, and adults [2–4]. Recent CT reports demonstrated that most of the lesions were distributed peripherally in the lung, which facilitates detection by lung ultrasound [5, 6]. In this study, we characterize the lung ultrasound findings COVID-19 pneumonia, and study the relationship between the ultrasound findings and clinical severity and the time-course of disease progress. Bedside lung ultrasound was performed to detect B-lines, lung consolidation, and pleural line abnormalities at 5 areas in each lung. Vascular ultrasound was also performed to detect potential deep vein thrombosis.

A total of 20 patients of COVID-19 pneumonia (12 males and 8 females) were categorized as 4 moderate, 5 severe, and 11 critical cases according to the current diagnosis and treatment program. All patients showed abnormal lung ultrasound findings, including 100% (20) pleural line abnormalities, 100% (20) B-lines, and 50% (10) consolidation. Most of the moderate and severe cases could show both separated B-lines and confluent B-lines during admission. All critical patients showed confluent B-lines, and 18% (2) of them had compact B-lines. Bilateral involvement was observed in all patients. The predominate involved areas in moderate patients were on the back, i.e., the interscapular and infrascapular areas. For severe and critical patients, all 5 areas could be involved. Consolidations were not detected in moderate cases, and distributed mainly on the posterior areas in severe and critical cases. Pleural effusion (18%, 2 cases), pericardial effusion (9%, 1 case), and deep vein thrombosis (64%, 5 cases) were only found in critical patients. (Table 1).

**Table 1:** Lung ultrasound findings in different clinical severity

	**Moderate (*n*** **= 4)**	**Severe (*n*** **= 5)**	**Critical (*n*** **= 11)**
**Pleural line abnormalities**	100% (4)	100% (5)	100% (11)
**B-lines**	100% (4)	100% (5)	100% (11)
Separated B-lines	75% (3)	80% (4)	36% (4)
Confluent B-lines	75% (3)	80% (4)	100% (11)
Compact B-lines	0	0	18% (2)
Bilateral involvement	100% (4)	100% (5)	100% (11)
Anterior upper area	25% (1)	100% (5)	100% (11)
Anterior lower area	50% (2)	100% (5)	100% (11)
Lateral area	75% (3)	100% (5)	100% (11)
Interscapular area	100% (4)	100% (5)	100% (11)
Infrascapular area	100% (4)	100% (5)	100% (11)
**Consolidation**	0	60% (3)	64% (7)
Bilateral involvement	0	40% (2)	54% (6)
Anterior upper area	0	0	18% (2)
Anterior lower area	0	0	18% (2)
Lateral area	0	20% (1)	18% (2)
Interscapular area	0	20% (1)	64% (7)
Infrascapular area	0	60% (3)	64% (7)
**Pleural effusion**	0	0	18% (2)
**Pericardial effusion**	0	0	9% (1)
**Deep vein thrombosis**	0	0	64% (7)

Data are presented as percentages (number of cases)

A total of 36 ultrasound examinations were categorized to four groups based on the time interval between onset of symptoms and ultrasound examinations (1st to 4th week). All of the examinations showed abnormal lung ultrasound findings, including 100% (36) pleural line abnormalities, 100% (36) B-lines, and 64% (23) consolidation. The separate B-lines were found more than half of the examinations after the 2nd week. The majority of examinations during the 2nd and 3rd weeks showed confluent B-lines. The involvement of anterior areas with B-lines decreased along with the infected time course. The lateral and back areas were always involved in all stages for B-lines. Consolidations were found in more than half examinations after the 1st week. Most of consolidation lesions confined within unilateral lung except at the 2nd week. The anterior and lateral areas were not involved of consolidations during 1st and 4th week. Consolidations were always found in the interscapular and infrascapular areas. Pleural effusion was observed across all stages, but only in a few examinations. The deep vein thrombosis can be found since the 1st week and most prevalent at the 4th week (62.5%, 5 cases). (Table 2).

**Table 2:** Lung ultrasound findings in different infection stage

	**1st week (*n*** **= 4)**	**2nd week (*n*** **= 11)**	**3rd week (*n*** **= 13)**	**4th week (*n*** **= 8)**
**Clinical severity (%)**				
Moderate	0	18% (2)	15% (2)	12.5% (1)
Severe	25% (1)	18% (2)	23% (3)	25% (2)
Critical	75% (3)	64% (7)	62% (8)	62.5% (5)
**Pleural line abnormalities**	100% (4)	100% (11)	100% (13)	100% (8)
**B-lines**	100% (4)	100% (11)	100% (13)	100% (8)
Separated B-lines	50% (2)	46% (5)	69% (9)	75% (6)
Confluent B-lines	75% (3)	91% (10)	85% (11)	75% (6)
Compact B-lines	25% (1)	18% (2)	15% (2)	0
Bilateral involvement	100% (4)	100% (11)	100% (13)	87.5% (7)
Anterior upper area	100% (4)	91% (10)	85% (11)	87.5% (7)
Anterior lower area	100% (4)	91% (10)	77% (10)	75% (6)
Lateral area	100% (4)	100% (11)	100% (13)	100% (8)
Interscapular area	100% (4)	100% (11)	100% (13)	87.5% (7)
Infrascapular area	100% (4)	100% (11)	100% (13)	100% (8)
**Consolidation**	50% (2)	55% (6)	77% (10)	62.5% (5)
Bilateral involvement	25% (1)	55% (6)	36% (4)	25% (2)
Anterior upper area	0	9% (1)	0	0
Anterior lower area	0	9% (1)	8% (1)	0
Lateral area	0	18% (2)	8% (1)	0
Interscapular area	50% (2)	55% (6)	54% (7)	62.5% (5)
Infrascapular area	25% (1)	55% (6)	77% (10)	62.5% (5)
**Pleural effusion**	25% (1)	9% (1)	15% (2)	12.5% (1)
**Pericardial effusion**	0	9% (1)	8% (1)	12.5% (1)
**Deep vein thrombosis**	50% (2)	46% (5)	46% (6)	62.5% (5)

Data are presented as percentages (number of cases)

In summary, abnormal lung ultrasound findings, mainly B-lines, consolidation and pleural line abnormalities, were found in patients infected with COVID-19 pneumonia. Bilateral involvement was always observed with predominant distribution in the posterior part of the lungs. The composition of different density of B-lines and areas of consolidation showed parallel changes with the clinical severity. The extent of disease demonstrated by ultrasound findings seemed reaching the peak at the 2nd week and recovering gradually thereafter. Collectively, lung ultrasound could serve as a valuable tool for the detection and follow-up of lung lesions in COVID-19 pneumonia and also provide supplemental imaging information for current recommended radiological examination, with the advantages of radiation-free, flexibility, and cost effective.
